# (7*R*,8*S*,9*S*,12*S*)-1-(4-Chloro­benz­yloxy)-13,14-didehydro-12-hy­droxy-2,13-dimeth­oxy-*N*-methyl­morphinane

**DOI:** 10.1107/S160053681103443X

**Published:** 2011-08-27

**Authors:** Xing-Liang Zheng, Ning-Fei Jiang, Hong-Sheng Gao, Dan Luo, Ai-Shun Ding

**Affiliations:** a School of Chemistry and Biological Engineering, Changsha University of Science & Technology, Changsha 410114, People’s Republic of China

## Abstract

The title compound, C_26_H_30_ClNO_4_, a sinomenine derivative, has five six-membered rings, two of which are aromatic, with a dihedral angle of 34.13 (20)° between these. The N-containing ring and the fourth ring exhibit chair conformations, while the fifth ring approximates an envelope conformation. A single inter­molecular O—H⋯N hydrogen-bonding inter­action gives a one-dimensional chain structure which extends along the *a* axis. The absolute configuration for the mol­ecule has been determined.

## Related literature

For background on biological effects of sinomenine derivatives and other related compounds, see: Liu *et al.* (1994 ▶, 1996 ▶, 1997 ▶); Mark *et al.* (2003 ▶); Ye *et al.* (2004 ▶). For related structures, see: Li *et al.* (2009 ▶); Batterham *et al.* (1965 ▶).
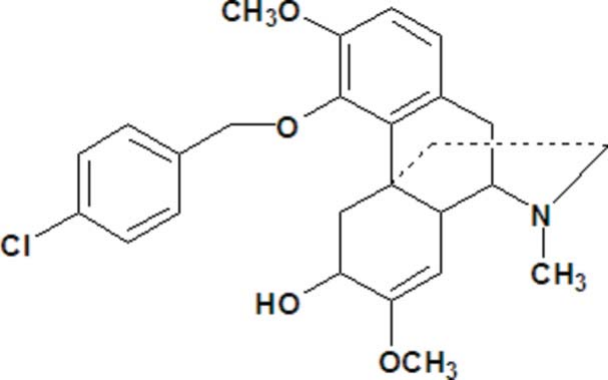


## Experimental

### 

#### Crystal data

C_26_H_30_ClNO_4_
*M* *_r_* = 455.96
Orthorhombic, 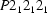

*a* = 7.8073 (9) Å
*b* = 9.7598 (11) Å
*c* = 31.043 (2) Å
*V* = 2365.4 (4) Å^3^
*Z* = 4
Mo *K*α radiation
μ = 0.19 mm^−1^
*T* = 296 K
0.37 × 0.31 × 0.26 mm

#### Data collection

Bruker SMART CCD area-detector diffractometer
Absorption correction: empirical (using intensity measurements) (*SADABS*; Bruker, 2000 ▶) *T*_min_ = 0.564, *T*_max_ = 1.000
12129 measured reflections
4590 independent reflections
3899 reflections with *I* > 2σ(*I*)
*R*_int_ = 0.035

#### Refinement

*R*[*F* ^2^ > 2σ(*F* ^2^)] = 0.042
*wR*(*F* ^2^) = 0.125
*S* = 1.07
4590 reflections
293 parameters
H-atom parameters constrained
Δρ_max_ = 0.21 e Å^−3^
Δρ_min_ = −0.20 e Å^−3^
Absolute structure: Flack, (1983 ▶), 1905 Friedel pairs
Flack parameter: 0.01 (8)

### 

Data collection: *SMART* (Bruker, 2000 ▶); cell refinement: *SAINT* (Bruker, 2000 ▶); data reduction: *SAINT*; program(s) used to solve structure: *SHELXS97* (Sheldrick, 2008 ▶); program(s) used to refine structure: *SHELXL97* (Sheldrick, 2008 ▶); molecular graphics: *SHELXTL* (Sheldrick, 2008 ▶); software used to prepare material for publication: *SHELXTL*.

## Supplementary Material

Crystal structure: contains datablock(s) global, I. DOI: 10.1107/S160053681103443X/zs2138sup1.cif

Structure factors: contains datablock(s) I. DOI: 10.1107/S160053681103443X/zs2138Isup2.hkl

Additional supplementary materials:  crystallographic information; 3D view; checkCIF report

## Figures and Tables

**Table 1:** Hydrogen-bond geometry (Å, °)

*D*—H⋯*A*	*D*—H	H⋯*A*	*D*⋯*A*	*D*—H⋯*A*
O3—H3⋯N1^i^	0.82	2.28	2.945 (2)	139

Symmetry code: (i) 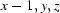.

**Fig. 1.**
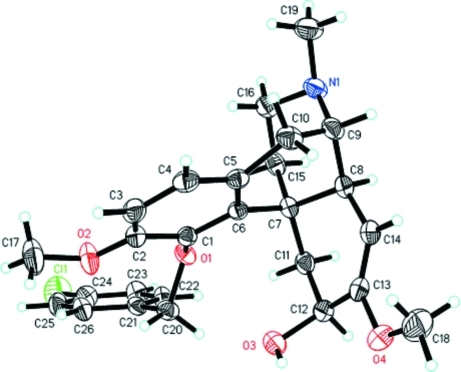
The molecular structure of the title compound showing 50% probability displacement ellipsoids.

**Fig. 2.**
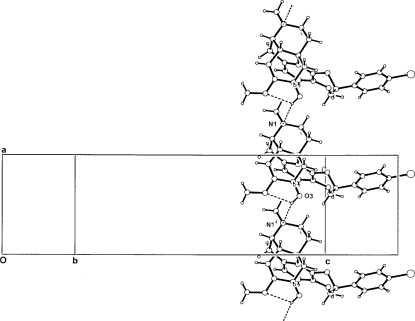
The one-dimensional hydrogen-bonded chain structure in the title compound, extending along the a axis of the unit cell. Hydrogen bonds are shown as dashed lines

## supplementary crystallographic information

### Comment

We have synthesized a new sinomenine derivative, the title compound 13,14-didehydro-1-(4'-chlorobenzyloxy)-*N*-methyl-2,13-\ dimethoxy-12-hydroxymorphinane, C_26_H_30_ClNO_4_ and report its crystal structure and its absolute confifuration. Biological effects of sinomenine derivatives and related compounds have been described (Liu *et al.*, 1994, 1996, 1997; Mark *et al.*, 2003; Ye *et al.*, 2004).

In the title compound (Fig. 1) there are two benzene planes, atoms C1/C2/C3/C4/C5/C6 form one plane and atoms C21···C26 form the second plane which has the *p*-chlorine substituent. The angle between these two planes is 34.14 (20)°. Ring *B* [C5···C10] in the molecule approximates an envelope conformation. In contrast, rings *D* [C7/C8/C9/N1/C16/C15] and *C* [C7/C11/C12/C13/C14/C8] exhibit almost regular chair conformations. Similar features have been described in related compounds (Li *et al.*, 2009; Batterham *et al.*, 1965). The absolute configuration in this chlorinated compound can be assigned as (C7*R*,C8*S*,C9*S*, C12*S*) for the four chiral centres in the molecule (using the trivial numbering system for the molecule). The crystal structure is stabilized by O—H···N hydrogen bonds (Table 1), linking the molecules into one- dimensional chains which extend along *a* (Fig. 2). An intramolecular hydroxyl O—H···O_methoxy_ interaction is also present. No significant aromatic π–π stacking interactions were found.

### Experimental

The title compound was obtained by reducing (9*S*,13*R*,14*S*)-7,8-\ didehydro-4-(4'-chlorobenzyloxy)-3,7-dimethoxy-17-methyl-morphinan-6-one with lithium aluminium tetrahydride. Colorless blocks (m.p. 412 K) were grown from an ethyl acetate–hexane solution.

### Refinement

H atoms were positioned geometrically, with C—H = 0.93 (aromatic CH), 0.96 (methyl CH_3_), 0.97 (methylene CH_2_) or 0.98 Å (methine CH), and were constrained to ride on their parent atoms, with *U*_iso_(H) = 1.2*U*_eq_(carrier C) or *U*_iso_(H) = 1.5*U*_eq_(carrier C). The absolute configuration for the molecule was assigned on the basis of the Flack parameter [0.01 (8)] determined using 1905 Friedel pairs.

### Crystal data

**Table:**

C_26_H_30_ClNO_4_	*D*_x_ = 1.280 Mg m^−^^3^
*M**_r_* = 455.96	Melting point: 412 K
Orthorhombic, *P*2_1_2_1_2_1_	Mo *K*α radiation, λ = 0.71073 Å
Hall symbol: P 2ac 2ab	Cell parameters from 3586 reflections
*a* = 7.8073 (9) Å	θ = 5.2–48.3°
*b* = 9.7598 (11) Å	µ = 0.19 mm^−^^1^
*c* = 31.043 (2) Å	*T* = 296 K
*V* = 2365.4 (4) Å^3^	Prismatic, colorless
*Z* = 4	0.37 × 0.31 × 0.26 mm
*F*(000) = 968	

### Data collection

**Table:**

Bruker SMART CCD area-detector diffractometer	4590 independent reflections
Radiation source: fine-focus sealed tube	3899 reflections with *I* > 2σ(*I*)
graphite	*R*_int_ = 0.035
φ and ω scans	θ_max_ = 26.0°, θ_min_ = 2.2°
Absorption correction: empirical (using intensity measurements) (*SADABS*; Bruker, 2000)	*h* = −9→9
*T*_min_ = 0.564, *T*_max_ = 1.000	*k* = −12→11
12129 measured reflections	*l* = −27→38

### Refinement

**Table:**

Refinement on *F*^2^	Secondary atom site location: difference Fourier map
Least-squares matrix: full	Hydrogen site location: inferred from neighbouring sites
*R*[*F*^2^ > 2σ(*F*^2^)] = 0.042	H-atom parameters constrained
*wR*(*F*^2^) = 0.125	*w* = 1/[σ^2^(*F*_o_^2^) + (0.0768*P*)^2^ + 0.0462*P*] where *P* = (*F*_o_^2^ + 2*F*_c_^2^)/3
*S* = 1.07	(Δ/σ)_max_ = 0.002
4590 reflections	Δρ_max_ = 0.21 e Å^−^^3^
293 parameters	Δρ_min_ = −0.20 e Å^−^^3^
0 restraints	Absolute structure: Flack, (1983), 1905 Friedel pairs
Primary atom site location: structure-invariant direct methods	Flack parameter: 0.01 (8)

### Special details

**Table:**

Geometry. All e.s.d.'s (except the e.s.d. in the dihedral angle between two l.s. planes) are estimated using the full covariance matrix. The cell e.s.d.'s are taken into account individually in the estimation of e.s.d.'s in distances, angles and torsion angles; correlations between e.s.d.'s in cell parameters are only used when they are defined by crystal symmetry. An approximate (isotropic) treatment of cell e.s.d.'s is used for estimating e.s.d.'s involving l.s. planes.
Refinement. Refinement of *F*^2^ against ALL reflections. The weighted *R*-factor *wR* and goodness of fit *S* are based on *F*^2^, conventional *R*-factors *R* are based on *F*, with *F* set to zero for negative *F*^2^. The threshold expression of *F*^2^ > σ(*F*^2^) is used only for calculating *R*-factors(gt) *etc*. and is not relevant to the choice of reflections for refinement. *R*-factors based on *F*^2^ are statistically about twice as large as those based on *F*, and *R*- factors based on ALL data will be even larger.

### Fractional atomic coordinates and isotropic or equivalent isotropic displacement parameters (Å^2^)

**Table:**

	*x*	*y*	*z*	*U*_iso_*/*U*_eq_	
Cl1	0.81142 (15)	0.78389 (10)	1.08775 (2)	0.1016 (3)	
N1	1.3185 (2)	0.1882 (2)	0.82782 (5)	0.0505 (4)	
O1	0.82630 (18)	0.38126 (14)	0.91976 (4)	0.0427 (3)	
O2	0.6757 (3)	0.18817 (19)	0.96710 (5)	0.0697 (5)	
O3	0.5912 (2)	0.39646 (19)	0.83107 (5)	0.0579 (4)	
H3	0.5190	0.3541	0.8173	0.087*	
O4	0.6195 (2)	0.3081 (2)	0.74526 (5)	0.0687 (5)	
C1	0.8194 (3)	0.2505 (2)	0.90402 (6)	0.0364 (4)	
C2	0.7464 (3)	0.1477 (2)	0.92900 (6)	0.0459 (5)	
C3	0.7542 (3)	0.0145 (2)	0.91506 (7)	0.0496 (5)	
H3A	0.6980	−0.0547	0.9301	0.060*	
C4	0.8467 (3)	−0.0151 (2)	0.87853 (7)	0.0471 (5)	
H4	0.8539	−0.1058	0.8696	0.056*	
C5	0.9287 (3)	0.0834 (2)	0.85473 (6)	0.0382 (4)	
C6	0.9078 (2)	0.22094 (19)	0.86587 (6)	0.0340 (4)	
C7	0.9998 (2)	0.3334 (2)	0.84059 (6)	0.0383 (4)	
C8	1.0587 (3)	0.2808 (2)	0.79620 (6)	0.0447 (5)	
H8	1.1328	0.3511	0.7835	0.054*	
C9	1.1650 (3)	0.1533 (2)	0.80212 (6)	0.0478 (5)	
H9	1.2048	0.1251	0.7735	0.057*	
C10	1.0486 (3)	0.0400 (2)	0.81902 (7)	0.0492 (5)	
H10A	0.9808	0.0050	0.7953	0.059*	
H10B	1.1195	−0.0345	0.8295	0.059*	
C11	0.8894 (3)	0.4595 (2)	0.83083 (7)	0.0464 (5)	
H11A	0.8539	0.5002	0.8579	0.056*	
H11B	0.9594	0.5264	0.8158	0.056*	
C12	0.7299 (3)	0.4310 (2)	0.80377 (6)	0.0511 (6)	
H12	0.6998	0.5159	0.7887	0.061*	
C13	0.7623 (3)	0.3232 (3)	0.77030 (6)	0.0512 (6)	
C14	0.9100 (3)	0.2596 (3)	0.76603 (6)	0.0508 (5)	
H14	0.9232	0.1985	0.7433	0.061*	
C15	1.1636 (3)	0.3704 (2)	0.86549 (6)	0.0464 (5)	
H15A	1.2238	0.4431	0.8505	0.056*	
H15B	1.1333	0.4037	0.8939	0.056*	
C16	1.2806 (3)	0.2468 (2)	0.86981 (6)	0.0482 (5)	
H16A	1.3865	0.2741	0.8837	0.058*	
H16B	1.2258	0.1782	0.8878	0.058*	
C17	0.6109 (5)	0.0904 (3)	0.99517 (9)	0.0903 (10)	
H17A	0.5093	0.0506	0.9831	0.135*	
H17B	0.5837	0.1328	1.0222	0.135*	
H17C	0.6951	0.0201	0.9997	0.135*	
C18	0.6254 (4)	0.2048 (4)	0.71317 (9)	0.0837 (9)	
H18A	0.7166	0.2244	0.6934	0.125*	
H18B	0.5185	0.2027	0.6979	0.125*	
H18C	0.6452	0.1175	0.7265	0.125*	
C19	1.4383 (3)	0.0751 (3)	0.83182 (9)	0.0670 (7)	
H19A	1.3955	0.0103	0.8524	0.100*	
H19B	1.5473	0.1093	0.8413	0.100*	
H19C	1.4514	0.0311	0.8044	0.100*	
C20	0.6655 (3)	0.4499 (2)	0.93041 (7)	0.0473 (5)	
H20A	0.5761	0.3830	0.9358	0.057*	
H20B	0.6295	0.5086	0.9069	0.057*	
C21	0.6997 (3)	0.5327 (2)	0.96992 (6)	0.0417 (5)	
C22	0.7929 (3)	0.6522 (2)	0.96719 (6)	0.0501 (5)	
H22	0.8317	0.6819	0.9405	0.060*	
C23	0.8294 (4)	0.7279 (2)	1.00317 (8)	0.0579 (6)	
H23	0.8939	0.8077	1.0009	0.070*	
C24	0.7706 (3)	0.6854 (3)	1.04222 (7)	0.0556 (6)	
C25	0.6774 (3)	0.5678 (3)	1.04635 (7)	0.0568 (6)	
H25	0.6379	0.5396	1.0732	0.068*	
C26	0.6428 (3)	0.4912 (2)	1.00989 (7)	0.0503 (5)	
H26	0.5803	0.4105	1.0124	0.060*	

### Atomic displacement parameters (Å^2^)

**Table:**

	*U*^11^	*U*^22^	*U*^33^	*U*^12^	*U*^13^	*U*^23^
Cl1	0.1384 (8)	0.0995 (7)	0.0670 (4)	−0.0144 (6)	−0.0017 (5)	−0.0405 (4)
N1	0.0360 (9)	0.0650 (12)	0.0504 (9)	0.0044 (9)	0.0017 (8)	−0.0077 (8)
O1	0.0412 (8)	0.0442 (8)	0.0428 (7)	−0.0012 (7)	0.0069 (6)	−0.0103 (6)
O2	0.0898 (13)	0.0707 (12)	0.0486 (8)	−0.0071 (11)	0.0288 (9)	0.0063 (8)
O3	0.0467 (9)	0.0767 (12)	0.0504 (8)	0.0009 (8)	0.0072 (7)	−0.0006 (8)
O4	0.0597 (10)	0.0942 (14)	0.0521 (9)	0.0099 (10)	−0.0120 (8)	0.0058 (9)
C1	0.0362 (10)	0.0376 (11)	0.0353 (8)	−0.0002 (8)	−0.0018 (7)	−0.0009 (7)
C2	0.0442 (11)	0.0527 (13)	0.0410 (10)	0.0005 (10)	0.0037 (9)	0.0067 (9)
C3	0.0481 (12)	0.0441 (13)	0.0567 (12)	−0.0054 (10)	−0.0003 (10)	0.0127 (9)
C4	0.0469 (12)	0.0353 (11)	0.0590 (12)	0.0041 (9)	−0.0052 (10)	0.0014 (9)
C5	0.0353 (10)	0.0389 (11)	0.0405 (9)	0.0022 (9)	−0.0065 (8)	−0.0037 (8)
C6	0.0318 (9)	0.0348 (10)	0.0354 (9)	−0.0008 (8)	−0.0024 (7)	−0.0003 (7)
C7	0.0406 (10)	0.0400 (11)	0.0344 (9)	−0.0022 (9)	0.0056 (8)	−0.0014 (8)
C8	0.0479 (12)	0.0514 (12)	0.0347 (9)	0.0018 (10)	0.0075 (8)	−0.0002 (8)
C9	0.0423 (11)	0.0612 (14)	0.0398 (10)	0.0053 (11)	0.0042 (9)	−0.0116 (9)
C10	0.0490 (13)	0.0458 (12)	0.0528 (11)	0.0050 (10)	−0.0006 (10)	−0.0138 (10)
C11	0.0537 (13)	0.0393 (11)	0.0461 (11)	0.0006 (9)	0.0106 (9)	0.0079 (9)
C12	0.0504 (13)	0.0565 (14)	0.0464 (11)	0.0095 (11)	0.0063 (10)	0.0130 (9)
C13	0.0528 (13)	0.0674 (15)	0.0334 (9)	0.0024 (11)	−0.0024 (9)	0.0101 (9)
C14	0.0542 (13)	0.0657 (15)	0.0325 (9)	0.0067 (11)	0.0025 (9)	−0.0025 (9)
C15	0.0458 (12)	0.0504 (12)	0.0432 (10)	−0.0130 (10)	0.0082 (9)	−0.0080 (9)
C16	0.0379 (11)	0.0624 (15)	0.0443 (10)	−0.0037 (10)	−0.0028 (8)	−0.0070 (10)
C17	0.110 (3)	0.090 (2)	0.0705 (18)	−0.012 (2)	0.0383 (17)	0.0208 (16)
C18	0.076 (2)	0.111 (3)	0.0644 (16)	−0.0101 (19)	−0.0194 (14)	−0.0050 (16)
C19	0.0493 (14)	0.0777 (18)	0.0739 (16)	0.0151 (13)	−0.0033 (12)	−0.0078 (14)
C20	0.0424 (12)	0.0517 (13)	0.0478 (11)	0.0064 (10)	0.0016 (9)	−0.0078 (9)
C21	0.0387 (11)	0.0451 (12)	0.0412 (10)	0.0050 (9)	0.0055 (8)	−0.0039 (8)
C22	0.0596 (14)	0.0470 (13)	0.0437 (10)	−0.0004 (11)	0.0109 (9)	0.0020 (9)
C23	0.0681 (15)	0.0427 (13)	0.0630 (13)	−0.0095 (12)	0.0086 (12)	−0.0064 (10)
C24	0.0656 (15)	0.0513 (14)	0.0498 (11)	0.0047 (12)	0.0006 (11)	−0.0128 (10)
C25	0.0714 (16)	0.0606 (15)	0.0384 (10)	0.0066 (13)	0.0112 (11)	0.0006 (9)
C26	0.0560 (14)	0.0447 (12)	0.0503 (11)	−0.0037 (10)	0.0140 (10)	−0.0006 (9)

### Geometric parameters (Å, °)

**Table:**

Cl1—C24	1.739 (4)	C11—H11A	0.9700
N1—C19	1.452 (4)	C11—H11B	0.9700
N1—C16	1.454 (4)	C12—C13	1.500 (4)
N1—C9	1.479 (4)	C12—H12	0.9800
O1—C1	1.367 (4)	C13—C14	1.317 (5)
O1—C20	1.461 (4)	C14—H14	0.9300
O2—C2	1.364 (4)	C15—C16	1.519 (5)
O2—C17	1.388 (4)	C15—H15A	0.9700
O3—C12	1.416 (4)	C15—H15B	0.9700
O3—H3	0.8200	C16—H16A	0.9700
O4—C13	1.367 (4)	C16—H16B	0.9700
O4—C18	1.418 (5)	C17—H17A	0.9600
C1—C2	1.390 (4)	C17—H17B	0.9600
C1—C6	1.401 (4)	C17—H17C	0.9600
C2—C3	1.372 (5)	C18—H18A	0.9600
C3—C4	1.375 (4)	C18—H18B	0.9600
C3—H3A	0.9300	C18—H18C	0.9600
C4—C5	1.371 (4)	C19—H19A	0.9600
C4—H4	0.9300	C19—H19B	0.9600
C5—C6	1.395 (5)	C19—H19C	0.9600
C5—C10	1.511 (4)	C20—C21	1.493 (4)
C6—C7	1.529 (4)	C20—H20A	0.9700
C7—C11	1.533 (4)	C20—H20B	0.9700
C7—C15	1.537 (4)	C21—C22	1.377 (5)
C7—C8	1.541 (4)	C21—C26	1.379 (4)
C8—C14	1.506 (4)	C22—C23	1.369 (4)
C8—C9	1.507 (5)	C22—H22	0.9300
C8—H8	0.9800	C23—C24	1.361 (4)
C9—C10	1.525 (5)	C23—H23	0.9300
C9—H9	0.9800	C24—C25	1.365 (5)
C10—H10A	0.9700	C25—C26	1.383 (4)
C10—H10B	0.9700	C25—H25	0.9300
C11—C12	1.528 (5)	C26—H26	0.9300
			
C19—N1—C16	110.7 (2)	C14—C13—O4	127.3 (3)
C19—N1—C9	113.2 (2)	C14—C13—C12	123.2 (2)
C16—N1—C9	114.1 (2)	O4—C13—C12	109.4 (2)
C1—O1—C20	118.33 (19)	C13—C14—C8	123.2 (2)
C2—O2—C17	119.5 (3)	C13—C14—H14	118.4
C12—O3—H3	109.5	C8—C14—H14	118.4
C13—O4—C18	116.7 (2)	C16—C15—C7	111.0 (2)
O1—C1—C2	119.4 (2)	C16—C15—H15A	109.4
O1—C1—C6	118.35 (17)	C7—C15—H15A	109.4
C2—C1—C6	121.7 (2)	C16—C15—H15B	109.4
O2—C2—C3	124.5 (2)	C7—C15—H15B	109.4
O2—C2—C1	116.2 (3)	H15A—C15—H15B	108.0
C3—C2—C1	119.3 (3)	N1—C16—C15	110.83 (19)
C2—C3—C4	118.8 (2)	N1—C16—H16A	109.5
C2—C3—H3A	120.6	C15—C16—H16A	109.5
C4—C3—H3A	120.6	N1—C16—H16B	109.5
C5—C4—C3	122.9 (2)	C15—C16—H16B	109.5
C5—C4—H4	118.6	H16A—C16—H16B	108.1
C3—C4—H4	118.6	O2—C17—H17A	109.5
C4—C5—C6	119.1 (2)	O2—C17—H17B	109.5
C4—C5—C10	119.2 (2)	H17A—C17—H17B	109.5
C6—C5—C10	121.59 (18)	O2—C17—H17C	109.5
C5—C6—C1	117.73 (17)	H17A—C17—H17C	109.5
C5—C6—C7	120.5 (2)	H17B—C17—H17C	109.5
C1—C6—C7	121.2 (2)	O4—C18—H18A	109.5
C6—C7—C11	114.44 (14)	O4—C18—H18B	109.5
C6—C7—C15	107.5 (2)	H18A—C18—H18B	109.5
C11—C7—C15	112.3 (2)	O4—C18—H18C	109.5
C6—C7—C8	111.1 (2)	H18A—C18—H18C	109.5
C11—C7—C8	105.0 (2)	H18B—C18—H18C	109.5
C15—C7—C8	106.2 (2)	N1—C19—H19A	109.5
C14—C8—C9	112.8 (2)	N1—C19—H19B	109.5
C14—C8—C7	111.8 (2)	H19A—C19—H19B	109.5
C9—C8—C7	109.30 (19)	N1—C19—H19C	109.5
C14—C8—H8	107.6	H19A—C19—H19C	109.5
C9—C8—H8	107.6	H19B—C19—H19C	109.5
C7—C8—H8	107.6	O1—C20—C21	106.3 (2)
N1—C9—C8	108.8 (2)	O1—C20—H20A	110.5
N1—C9—C10	117.6 (2)	C21—C20—H20A	110.5
C8—C9—C10	108.2 (3)	O1—C20—H20B	110.5
N1—C9—H9	107.2	C21—C20—H20B	110.5
C8—C9—H9	107.2	H20A—C20—H20B	108.7
C10—C9—H9	107.2	C22—C21—C26	118.3 (2)
C5—C10—C9	114.7 (2)	C22—C21—C20	120.2 (2)
C5—C10—H10A	108.6	C26—C21—C20	121.5 (3)
C9—C10—H10A	108.6	C23—C22—C21	121.1 (2)
C5—C10—H10B	108.6	C23—C22—H22	119.4
C9—C10—H10B	108.6	C21—C22—H22	119.4
H10A—C10—H10B	107.6	C24—C23—C22	119.5 (3)
C12—C11—C7	114.9 (2)	C24—C23—H23	120.3
C12—C11—H11A	108.5	C22—C23—H23	120.3
C7—C11—H11A	108.5	C23—C24—C25	121.3 (2)
C12—C11—H11B	108.5	C23—C24—Cl1	119.6 (3)
C7—C11—H11B	108.5	C25—C24—Cl1	119.1 (2)
H11A—C11—H11B	107.5	C24—C25—C26	118.8 (2)
O3—C12—C13	112.1 (2)	C24—C25—H25	120.6
O3—C12—C11	109.7 (3)	C26—C25—H25	120.6
C13—C12—C11	111.8 (2)	C21—C26—C25	121.0 (3)
O3—C12—H12	107.6	C21—C26—H26	119.5
C13—C12—H12	107.6	C25—C26—H26	119.5
C11—C12—H12	107.6		
			
C20—O1—C1—C2	−60.3 (3)	C7—C8—C9—C10	67.2 (2)
C20—O1—C1—C6	128.7 (2)	C4—C5—C10—C9	−165.7 (2)
C17—O2—C2—C3	2.2 (4)	C6—C5—C10—C9	10.2 (3)
C17—O2—C2—C1	−175.6 (3)	N1—C9—C10—C5	79.1 (3)
O1—C1—C2—O2	4.2 (3)	C8—C9—C10—C5	−44.6 (3)
C6—C1—C2—O2	174.93 (19)	C6—C7—C11—C12	−60.6 (3)
O1—C1—C2—C3	−173.7 (2)	C15—C7—C11—C12	176.50 (15)
C6—C1—C2—C3	−3.0 (3)	C8—C7—C11—C12	61.5 (2)
O2—C2—C3—C4	−172.3 (2)	C7—C11—C12—O3	88.6 (2)
C1—C2—C3—C4	5.4 (3)	C7—C11—C12—C13	−36.5 (2)
C2—C3—C4—C5	−1.5 (3)	C18—O4—C13—C14	5.5 (4)
C3—C4—C5—C6	−5.0 (3)	C18—O4—C13—C12	−177.0 (2)
C3—C4—C5—C10	171.0 (2)	O3—C12—C13—C14	−122.1 (3)
C4—C5—C6—C1	7.2 (3)	C11—C12—C13—C14	1.6 (3)
C10—C5—C6—C1	−168.65 (18)	O3—C12—C13—O4	60.2 (3)
C4—C5—C6—C7	178.88 (18)	C11—C12—C13—O4	−176.05 (17)
C10—C5—C6—C7	3.0 (3)	O4—C13—C14—C8	−178.3 (2)
O1—C1—C6—C5	167.44 (18)	C12—C13—C14—C8	4.4 (4)
C2—C1—C6—C5	−3.4 (3)	C9—C8—C14—C13	147.1 (2)
O1—C1—C6—C7	−4.2 (3)	C7—C8—C14—C13	23.4 (3)
C2—C1—C6—C7	−175.01 (19)	C6—C7—C15—C16	60.3 (2)
C5—C6—C7—C11	137.6 (2)	C11—C7—C15—C16	−172.96 (16)
C1—C6—C7—C11	−51.1 (3)	C8—C7—C15—C16	−58.7 (2)
C5—C6—C7—C15	−97.0 (3)	C19—N1—C16—C15	176.63 (18)
C1—C6—C7—C15	74.4 (3)	C9—N1—C16—C15	−54.3 (3)
C5—C6—C7—C8	18.9 (2)	C7—C15—C16—N1	54.9 (2)
C1—C6—C7—C8	−169.73 (18)	C1—O1—C20—C21	142.79 (17)
C6—C7—C8—C14	71.4 (2)	O1—C20—C21—C22	74.5 (3)
C11—C7—C8—C14	−52.8 (2)	O1—C20—C21—C26	−104.1 (3)
C15—C7—C8—C14	−171.90 (18)	C26—C21—C22—C23	0.3 (3)
C6—C7—C8—C9	−54.2 (3)	C20—C21—C22—C23	−178.3 (2)
C11—C7—C8—C9	−178.37 (17)	C21—C22—C23—C24	−0.9 (4)
C15—C7—C8—C9	62.5 (3)	C22—C23—C24—C25	0.8 (4)
C19—N1—C9—C8	−174.31 (18)	C22—C23—C24—Cl1	−178.1 (2)
C16—N1—C9—C8	57.9 (2)	C23—C24—C25—C26	−0.1 (4)
C19—N1—C9—C10	62.2 (3)	Cl1—C24—C25—C26	178.9 (2)
C16—N1—C9—C10	−65.6 (3)	C22—C21—C26—C25	0.4 (4)
C14—C8—C9—N1	173.26 (16)	C20—C21—C26—C25	179.0 (2)
C7—C8—C9—N1	−61.7 (2)	C24—C25—C26—C21	−0.5 (4)
C14—C8—C9—C10	−57.8 (3)		

### Hydrogen-bond geometry (Å, °)

**Table:**

*D*—H···*A*	*D*—H	H···*A*	*D*···*A*	*D*—H···*A*
O3—H3···N1^i^	0.82	2.28	2.945 (2)	139
O3—H3···O4	0.82	2.41	2.809 (2)	111

Symmetry codes: (i) *x*−1, *y*, *z*.
